# 2-(4-{3-[1-(3-Bromo­prop­yl)-3,3-dimethyl-2,3-dihydro-1*H*-indol-2-yl­idene]prop-1-en­yl}-3-cyano-5,5-dimethyl-2,5-dihydro­furan-2-yl­idene)malononitrile

**DOI:** 10.1107/S1600536809017747

**Published:** 2009-05-20

**Authors:** Graeme J. Gainsford, M. Delower H. Bhuiyan, Andrew J. Kay

**Affiliations:** a Industrial Research Limited, PO Box 31-310, Lower Hutt, New Zealand

## Abstract

The backbone of the title mol­ecule, C_26_H_25_BrN_4_O, is approximately planar: the dihedral angle between the planes of the indoline ring system and the furan ring is 7.68 (14)°. In the crystal, layers lying parallel to (10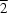) occur, with the mol­ecules inter­acting *via* weak C—H⋯N(cyano) and C—H⋯Br bonds and short N(cyano)⋯Br contacts [3.345 (4) Å].

## Related literature

For general background to zwitterionic dyes and their applications, see: Dalton (2002 ▶); Gainsford *et al.* (2007 ▶, 2008 ▶); Kay *et al.* (2004 ▶). For related structures, see: Li *et al.* (2005 ▶); Marder *et al.* (1993 ▶); Mushkalo & Sogulayaev (1986 ▶); Wang *et al.* (2007 ▶). For a description of the Cambridge Stuctural Database, see: Allen (2002 ▶).
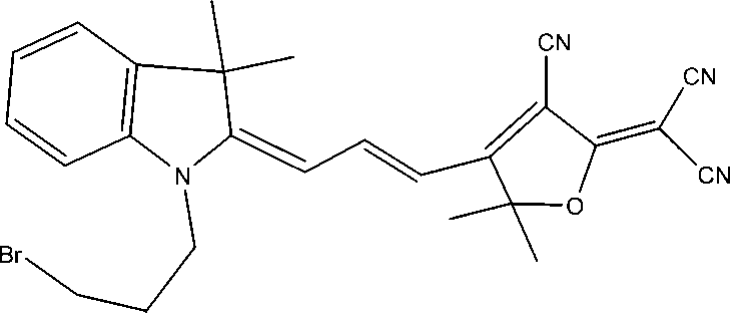


## Experimental

### 

#### Crystal data

C_26_H_25_BrN_4_O
*M* *_r_* = 489.41
Monoclinic, 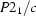

*a* = 10.2349 (4) Å
*b* = 9.4017 (4) Å
*c* = 24.4524 (10) Å
β = 96.175 (2)°
*V* = 2339.29 (17) Å^3^
*Z* = 4
Mo *K*α radiation
μ = 1.78 mm^−1^
*T* = 122 K
0.85 × 0.36 × 0.10 mm

#### Data collection

Bruker APEXII CCD diffractometer
Absorption correction: multi-scan (Blessing, 1995 ▶) *T*_min_ = 0.549, *T*_max_ = 0.746 (expected range = 0.616–0.837)
56895 measured reflections
6791 independent reflections
5482 reflections with *I* > 2σ(*I*)
*R*_int_ = 0.044

#### Refinement

*R*[*F* ^2^ > 2σ(*F* ^2^)] = 0.059
*wR*(*F* ^2^) = 0.156
*S* = 1.19
6791 reflections
293 parameters
H-atom parameters constrained
Δρ_max_ = 3.08 e Å^−3^
Δρ_min_ = −0.60 e Å^−3^

### 

Data collection: *APEX2* (Bruker, 2005 ▶); cell refinement: *SAINT* (Bruker, 2005 ▶); data reduction: *SAINT* and *SADABS* (Bruker, 2005 ▶); program(s) used to solve structure: *SHELXS97* (Sheldrick, 2008 ▶); program(s) used to refine structure: *SHELXL97* (Sheldrick, 2008 ▶); molecular graphics: *ORTEP-3* (Farrugia, 1997 ▶), *PLATON* (Spek, 2009 ▶) and *Mercury* (Macrae *et al.*, 2006 ▶); software used to prepare material for publication: *SHELXL97* and *PLATON*.

## Supplementary Material

Crystal structure: contains datablocks global, I. DOI: 10.1107/S1600536809017747/hb2973sup1.cif

Structure factors: contains datablocks I. DOI: 10.1107/S1600536809017747/hb2973Isup2.hkl

Additional supplementary materials:  crystallographic information; 3D view; checkCIF report

## Figures and Tables

**Table 1:** Hydrogen-bond geometry (Å, °)

*D*—H⋯*A*	*D*—H	H⋯*A*	*D*⋯*A*	*D*—H⋯*A*
C9—H9*B*⋯N1^i^	0.98	2.59	3.449 (5)	147
C23—H23*B*⋯Br1^ii^	0.98	2.99	3.962 (4)	171
C26—H26*B*⋯Br1^iii^	0.99	2.95	3.815 (4)	147

Symmetry codes: (i) 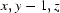; (ii) 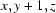; (iii) 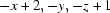.

**Fig. 1.**
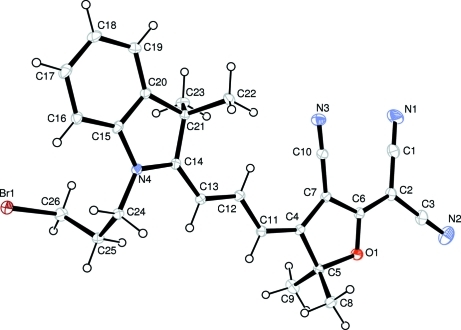
Molecular structure of the asymmetric unit (Farrugia, 1997); displacement ellipsoids are shown at the 30% probability level.

**Fig. 2.**
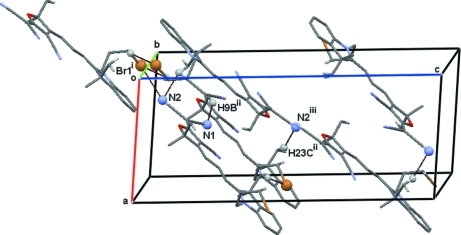
Packing diagram of the unit cell. Contact atoms are shown as balls; not all interactions and labels are shown for clarity (see text). Symmetry (i) x - 1,1/2 - y, z - 1/2 (ii) x, 1 + y, z (iii) 1 - x, y - 1/2,1/2 - z.

## supplementary crystallographic information

### Comment

The X-ray crystallographic and structural properties of zwitterionic dyes and their precursors have been a subject of some interest to us (Gainsford *et al.*, 2007, 2008) due to their potential application in a number of photonic and optoelectronic devices (Dalton, 2002; Kay *et al.*, 2004). The title compound was unintentionally synthesized en route to 2-{3-Cyano-4-[2-(10,10-dimethyl-6,7,8,10-tetrahydro-pyrido[1,2-*a*] indol-9-yl)-vinyl]-5,5-dimethyl-5*H*-furan-2-ylidene}-malononitrile. Compound REFCODES are from the C.S.D. (Version 5.30, with February 2009 updates; Allen, 2002)

The asymmetric unit contents are shown in Figure 1. The 5-membered ring plane of atoms O1,C4—C7 (hereafter "CDFP", [3-Cyano-5,5-Dimethyl-2,5-dihydrofuran-2-ylidene]propanedinitrile) can also be regarded as planar in this case (r.m.s. deviations 0.024 (3) Å). The dicyano group (N1,C1,C2,C3,N2) is planar (r.m.s.d. 0.008 (3) Å) but twisted by 6.6 (2)° with respect to the "CDFP" group; this is similar to the twist in related compound NOJKUT (Gainsford *et al.*, 2008) of 5.69 (17)°. The fused indolylidene system (atoms N4, C14 to C21) is also essentially planar (r.m.s.d. 0.017 (3) Å) and makes a dihedral angle with the "CDFP" ring of 7.68 (14)°. This reflects a twist in the C11–C14 polyene chain beginning at C11: the plane through C11–C14 subtends 5.4 (3)° with the "CDFP" plane. There is considerable delocalization of charge along the polyene /"CDFP" chain with a bond length alternation (BLA) value (Marder *et al.*, 1993) of 0.012Å compared with the free "CDFP" value of 0.108Å (Li *et al.*, 2005) and 0.060Å in GIMQAV (Gainsford *et al.*, 2007).

The almost planar molecules are arranged into nearly coplanar layers parallel to the (1,0,-2) plane with only *C*–*H*···*N*(cyano), C–H···Br and *N*(cyano)···Br contacts. The (methyl)*C*–*H*···*N*(cyano) contact (Table 1) is similar to that observed in several structures (Allen, 2002), where the methyl group is constrained by other interactions *e.g.* in JETGEV (Wang *et al.* 2007; N···H 2.57 Å, C–H···N 157°) the cyano nitrogen involved is bifurcated by a polyene C–H···N interation (H···N 2.72 Å, C–H···N 157°). Here the distance to the equivalent polyene H (H11) is 2.75 Å, with C–H···N 161°. In NOJKUT, a similar interaction is observed: H···N 2.45 Å, C–H···N 156°. The bromine atoms provide weak linking interactions: N2···Br1 3.345 (4)Å (Br1 at *x* - 1,1/2 - *y*,*z* - 1/2) and two C–H···Br interactions (Table 1). A final interaction is noted for completeness that would complete a weak interacting chain (N2···H23C(C23)H23B···Br1···N2) with H23C···N2 (N2 at 1 - *x*, *y* - 1/2,1/2 - *z*) and provide a weak interplanar link (see also Figure 2).

### Experimental

A mixture of 1 g (2.77 mmol) of 1-(3-bromopropyl)-2,3,3-trimethyl-3*H*-indolium bromide (Mushkalo & Sogulayaev, 1986), 883 mg (2.21 mmol) of {4-(2-acetanilidoethenyl)-3-cyano-5,5-dimethyl-2(5*H*)-furanylidene} propanedinitrile (compound 11*a*; Kay *et al.*, 2004) and triethylamine as a catalyst in 30 ml me thanol was refluxed for 3 h. After cooling to room temperature, the precipitate was filtered and washed with copious quantities of hot water, followed by small portions of cold methanol to afford the target molecule as a red-purple powder (720 mg, 67%). Platy crystals, of limited quality, were grown from a 2:1 dichloromethane/hexanes mixture. Mp: 264–266 °C; ^1^H NMR (500 MHz, CDCl_3_) δ 1.61 (6 H, s, 2 *x* CH_3_), 1.72 (6 H, s, 2 *x* CH_3_), 2.34 (2 H, qn, CH_2_), 3.50 (2 H, t, *J* 5.7 Hz, CH_2_), 4.06 (2 H, t, *J* 7.2 Hz, CH_2_), 5.78 (1 H, d, *J* 12.9 Hz, CH), 5.85 (1 H, d, *J* 12.9 Hz, CH), 7.04 (1 H, d, *J* 7.8 Hz, ArH), 7.16–7.21 (1 H, m, ArH), 7.31–7.37 (2 H, m, ArH), 8.78 (1*H*, br s, ArH); ^13^C NMR (125 MHz, CDCl_3_) δ 26.4, 27.7, 29.8, 29.9, 41.8, 48.9, 95.7, 99.9, 107.3, 109.3, 112.9, 113.8, 122.4, 124.6, 128.6, 140.4, 142.0, 147.3, 172.7, 177.4.

### Refinement

The final residual map peak is 1.19Å from Br1. On the basis of average I/σ(I) analysis, data was excluded for θ > 30°. Four reflections affected by the backstop and 19 others which were clearly outlier data presumably affected by residual material (with F_o_ >>F_c_ and Δ(F_o_^2^)/σ(F_o_^2^) > 4.9) were omitted from the refinements (using *OMIT*). All methyl and tertiary H atoms were refined with *U*_iso_ 1.5 & 1.2 times respectively that of the *U*_eq_ of their parent atom. All H atoms bound to carbon were constrained to their expected geometries (C—H 0.95, 0.98 & 1.00 Å).

### Crystal data

**Table:**

C_26_H_25_BrN_4_O	*F*(000) = 1008
*M**_r_* = 489.41	*D*_x_ = 1.390 Mg m^−^^3^
Monoclinic, *P*2_1_/*c*	Mo *K*α radiation, λ = 0.71073 Å
Hall symbol: -P 2ybc	Cell parameters from 9973 reflections
*a* = 10.2349 (4) Å	θ = 2.3–29.3°
*b* = 9.4017 (4) Å	µ = 1.78 mm^−^^1^
*c* = 24.4524 (10) Å	*T* = 122 K
β = 96.175 (2)°	Block, red
*V* = 2339.29 (17) Å^3^	0.85 × 0.36 × 0.10 mm
*Z* = 4	

### Data collection

**Table:**

Bruker APEXII CCD diffractometer	6791 independent reflections
Radiation source: fine-focus sealed tube	5482 reflections with *I* > 2σ(*I*)
graphite	*R*_int_ = 0.044
Detector resolution: 8.333 pixels mm^-1^	θ_max_ = 30.0°, θ_min_ = 2.5°
φ and ω scans	*h* = −14→14
Absorption correction: multi-scan (Blessing, 1995)	*k* = −13→13
*T*_min_ = 0.549, *T*_max_ = 0.746	*l* = −34→34
56895 measured reflections	

### Refinement

**Table:**

Refinement on *F*^2^	Primary atom site location: structure-invariant direct methods
Least-squares matrix: full	Secondary atom site location: difference Fourier map
*R*[*F*^2^ > 2σ(*F*^2^)] = 0.059	Hydrogen site location: inferred from neighbouring sites
*wR*(*F*^2^) = 0.156	H-atom parameters constrained
*S* = 1.19	*w* = 1/[σ^2^(*F*_o_^2^) + (0.0596*P*)^2^ + 4.5665*P*] where *P* = (*F*_o_^2^ + 2*F*_c_^2^)/3
6791 reflections	(Δ/σ)_max_ = 0.001
293 parameters	Δρ_max_ = 3.08 e Å^−^^3^
0 restraints	Δρ_min_ = −0.60 e Å^−^^3^

### Special details

**Table:**

Geometry. All e.s.d.'s (except the e.s.d. in the dihedral angle between two l.s. planes) are estimated using the full covariance matrix. The cell e.s.d.'s are taken into account individually in the estimation of e.s.d.'s in distances, angles and torsion angles; correlations between e.s.d.'s in cell parameters are only used when they are defined by crystal symmetry. An approximate (isotropic) treatment of cell e.s.d.'s is used for estimating e.s.d.'s involving l.s. planes.
Refinement. Refinement of *F*^2^ against ALL reflections. The weighted *R*-factor *wR* and goodness of fit *S* are based on *F*^2^, conventional *R*-factors *R* are based on *F*, with *F* set to zero for negative *F*^2^. The threshold expression of *F*^2^ > σ(*F*^2^) is used only for calculating *R*-factors(gt) *etc*. and is not relevant to the choice of reflections for refinement. *R*-factors based on *F*^2^ are statistically about twice as large as those based on *F*, and *R*- factors based on ALL data will be even larger.

### Fractional atomic coordinates and isotropic or equivalent isotropic displacement parameters (Å^2^)

**Table:**

	*x*	*y*	*z*	*U*_iso_*/*U*_eq_	
Br1	1.04264 (4)	−0.19667 (4)	0.464951 (13)	0.03123 (11)	
O1	0.4789 (2)	0.4651 (2)	0.11618 (9)	0.0259 (5)	
N1	0.5733 (4)	0.9332 (3)	0.18253 (17)	0.0487 (9)	
N2	0.3317 (4)	0.7471 (5)	0.04014 (15)	0.0487 (9)	
N3	0.7924 (3)	0.6919 (3)	0.24466 (12)	0.0302 (6)	
N4	1.0399 (2)	0.1410 (3)	0.36430 (10)	0.0183 (4)	
C1	0.5374 (4)	0.8345 (4)	0.15816 (16)	0.0330 (7)	
C2	0.4909 (3)	0.7121 (3)	0.12784 (14)	0.0258 (6)	
C3	0.4020 (3)	0.7300 (4)	0.07938 (15)	0.0317 (7)	
C4	0.6310 (3)	0.3831 (3)	0.18773 (11)	0.0186 (5)	
C5	0.5308 (3)	0.3319 (3)	0.14217 (12)	0.0210 (6)	
C6	0.5325 (3)	0.5769 (3)	0.14380 (12)	0.0210 (5)	
C7	0.6286 (3)	0.5313 (3)	0.18679 (12)	0.0195 (5)	
C8	0.5913 (3)	0.2449 (4)	0.09903 (13)	0.0263 (6)	
H8A	0.5238	0.2225	0.0688	0.040*	
H8B	0.6274	0.1564	0.1156	0.040*	
H8C	0.6619	0.2997	0.0849	0.040*	
C9	0.4168 (3)	0.2573 (4)	0.16424 (16)	0.0308 (7)	
H9A	0.3757	0.3215	0.1890	0.046*	
H9B	0.4486	0.1720	0.1845	0.046*	
H9C	0.3520	0.2300	0.1336	0.046*	
C10	0.7165 (3)	0.6235 (3)	0.21904 (12)	0.0213 (5)	
C11	0.7057 (3)	0.2857 (3)	0.22079 (12)	0.0208 (5)	
H11	0.6935	0.1877	0.2123	0.025*	
C12	0.7964 (3)	0.3200 (3)	0.26505 (12)	0.0213 (6)	
H12	0.8067	0.4170	0.2755	0.026*	
C13	0.8724 (3)	0.2188 (3)	0.29458 (12)	0.0209 (5)	
H13	0.8588	0.1224	0.2838	0.025*	
C14	0.9674 (3)	0.2454 (3)	0.33870 (11)	0.0182 (5)	
C15	1.1325 (3)	0.1961 (3)	0.40603 (11)	0.0201 (5)	
C16	1.2270 (3)	0.1236 (3)	0.43992 (12)	0.0214 (5)	
H16	1.2363	0.0233	0.4377	0.026*	
C17	1.3078 (3)	0.2043 (4)	0.47734 (13)	0.0280 (6)	
H17	1.3740	0.1583	0.5012	0.034*	
C18	1.2933 (3)	0.3511 (4)	0.48045 (14)	0.0293 (7)	
H18	1.3500	0.4040	0.5063	0.035*	
C19	1.1968 (3)	0.4214 (4)	0.44613 (13)	0.0267 (6)	
H19	1.1860	0.5215	0.4485	0.032*	
C20	1.1171 (3)	0.3418 (3)	0.40854 (12)	0.0203 (5)	
C21	1.0071 (3)	0.3868 (3)	0.36571 (11)	0.0193 (5)	
C22	1.0602 (3)	0.4925 (3)	0.32554 (14)	0.0272 (6)	
H22A	1.1278	0.4459	0.3064	0.041*	
H22B	1.0984	0.5748	0.3460	0.041*	
H22C	0.9882	0.5241	0.2986	0.041*	
C23	0.8931 (3)	0.4525 (4)	0.39357 (13)	0.0263 (6)	
H23A	0.8224	0.4806	0.3654	0.039*	
H23B	0.9248	0.5363	0.4148	0.039*	
H23C	0.8596	0.3823	0.4182	0.039*	
C24	1.0321 (3)	−0.0092 (3)	0.34754 (12)	0.0206 (5)	
H24A	1.1117	−0.0592	0.3643	0.025*	
H24B	1.0317	−0.0147	0.3071	0.025*	
C25	0.9108 (3)	−0.0860 (3)	0.36403 (12)	0.0237 (6)	
H25A	0.8316	−0.0389	0.3455	0.028*	
H25B	0.9116	−0.1849	0.3502	0.028*	
C26	0.8990 (3)	−0.0903 (4)	0.42482 (13)	0.0281 (6)	
H26A	0.8143	−0.1349	0.4311	0.034*	
H26B	0.8990	0.0082	0.4392	0.034*	

### Atomic displacement parameters (Å^2^)

**Table:**

	*U*^11^	*U*^22^	*U*^33^	*U*^12^	*U*^13^	*U*^23^
Br1	0.0428 (2)	0.02650 (17)	0.02325 (16)	−0.00417 (14)	−0.00169 (12)	0.00277 (13)
O1	0.0256 (10)	0.0217 (10)	0.0283 (11)	0.0037 (8)	−0.0076 (8)	0.0013 (9)
N1	0.061 (2)	0.0208 (15)	0.061 (2)	0.0085 (15)	−0.0063 (18)	−0.0004 (15)
N2	0.0389 (18)	0.065 (2)	0.0400 (19)	0.0197 (17)	−0.0053 (15)	0.0059 (17)
N3	0.0309 (14)	0.0266 (14)	0.0326 (14)	0.0013 (11)	0.0015 (11)	−0.0061 (12)
N4	0.0171 (10)	0.0198 (11)	0.0173 (11)	−0.0004 (9)	−0.0010 (8)	0.0007 (9)
C1	0.0345 (17)	0.0250 (16)	0.0390 (19)	0.0095 (13)	0.0019 (14)	0.0068 (14)
C2	0.0247 (14)	0.0243 (15)	0.0280 (15)	0.0087 (12)	0.0012 (11)	0.0049 (12)
C3	0.0285 (16)	0.0319 (18)	0.0348 (18)	0.0122 (13)	0.0032 (13)	0.0056 (14)
C4	0.0176 (12)	0.0212 (13)	0.0169 (12)	0.0015 (10)	0.0013 (9)	−0.0016 (10)
C5	0.0188 (12)	0.0187 (13)	0.0242 (14)	0.0030 (10)	−0.0044 (10)	0.0021 (10)
C6	0.0196 (12)	0.0221 (14)	0.0210 (13)	0.0046 (10)	0.0017 (10)	0.0015 (11)
C7	0.0198 (12)	0.0193 (13)	0.0193 (13)	0.0032 (10)	0.0012 (10)	−0.0007 (10)
C8	0.0299 (16)	0.0261 (15)	0.0221 (14)	0.0014 (12)	−0.0016 (12)	−0.0022 (12)
C9	0.0172 (13)	0.0291 (16)	0.046 (2)	0.0003 (12)	0.0014 (13)	0.0062 (14)
C10	0.0228 (13)	0.0179 (13)	0.0232 (14)	0.0042 (10)	0.0034 (10)	−0.0013 (11)
C11	0.0215 (13)	0.0190 (13)	0.0212 (13)	0.0018 (10)	−0.0016 (10)	0.0005 (10)
C12	0.0209 (13)	0.0216 (14)	0.0210 (13)	0.0000 (10)	0.0004 (10)	−0.0001 (10)
C13	0.0226 (13)	0.0192 (13)	0.0200 (13)	−0.0012 (10)	−0.0021 (10)	0.0001 (10)
C14	0.0178 (12)	0.0204 (13)	0.0165 (12)	0.0000 (10)	0.0018 (10)	0.0025 (10)
C15	0.0159 (11)	0.0288 (14)	0.0155 (12)	−0.0019 (11)	0.0017 (9)	0.0028 (11)
C16	0.0188 (12)	0.0238 (14)	0.0212 (13)	−0.0010 (10)	0.0002 (10)	0.0026 (11)
C17	0.0198 (13)	0.0391 (18)	0.0237 (14)	−0.0021 (13)	−0.0043 (11)	0.0043 (13)
C18	0.0241 (14)	0.0344 (17)	0.0271 (15)	−0.0080 (13)	−0.0074 (12)	−0.0015 (13)
C19	0.0272 (14)	0.0232 (15)	0.0283 (15)	−0.0074 (12)	−0.0040 (12)	0.0001 (12)
C20	0.0194 (12)	0.0221 (13)	0.0188 (13)	−0.0042 (10)	−0.0008 (10)	0.0024 (10)
C21	0.0201 (12)	0.0191 (13)	0.0179 (12)	−0.0038 (10)	−0.0011 (10)	0.0024 (10)
C22	0.0296 (15)	0.0215 (14)	0.0296 (15)	−0.0067 (12)	−0.0014 (12)	0.0065 (12)
C23	0.0253 (14)	0.0252 (15)	0.0278 (15)	−0.0004 (12)	0.0009 (11)	−0.0057 (12)
C24	0.0237 (13)	0.0194 (13)	0.0184 (13)	0.0011 (10)	0.0012 (10)	−0.0014 (10)
C25	0.0253 (14)	0.0222 (14)	0.0227 (14)	−0.0060 (11)	−0.0021 (11)	−0.0002 (11)
C26	0.0278 (15)	0.0312 (17)	0.0261 (15)	−0.0029 (13)	0.0061 (12)	0.0006 (13)

### Geometric parameters (Å, °)

**Table:**

Br1—C26	1.953 (3)	C13—H13	0.9500
O1—C6	1.336 (4)	C14—C21	1.520 (4)
O1—C5	1.476 (3)	C15—C20	1.382 (4)
N1—C1	1.142 (5)	C15—C16	1.383 (4)
N2—C3	1.147 (5)	C16—C17	1.390 (4)
N3—C10	1.142 (4)	C16—H16	0.9500
N4—C14	1.344 (4)	C17—C18	1.392 (5)
N4—C15	1.414 (4)	C17—H17	0.9500
N4—C24	1.470 (4)	C18—C19	1.393 (4)
C1—C2	1.423 (5)	C18—H18	0.9500
C2—C6	1.383 (4)	C19—C20	1.382 (4)
C2—C3	1.424 (5)	C19—H19	0.9500
C4—C7	1.394 (4)	C20—C21	1.513 (4)
C4—C11	1.395 (4)	C21—C22	1.537 (4)
C4—C5	1.510 (4)	C21—C23	1.542 (4)
C5—C9	1.510 (4)	C22—H22A	0.9800
C5—C8	1.519 (4)	C22—H22B	0.9800
C6—C7	1.426 (4)	C22—H22C	0.9800
C7—C10	1.424 (4)	C23—H23A	0.9800
C8—H8A	0.9800	C23—H23B	0.9800
C8—H8B	0.9800	C23—H23C	0.9800
C8—H8C	0.9800	C24—C25	1.528 (4)
C9—H9A	0.9800	C24—H24A	0.9900
C9—H9B	0.9800	C24—H24B	0.9900
C9—H9C	0.9800	C25—C26	1.505 (4)
C11—C12	1.386 (4)	C25—H25A	0.9900
C11—H11	0.9500	C25—H25B	0.9900
C12—C13	1.383 (4)	C26—H26A	0.9900
C12—H12	0.9500	C26—H26B	0.9900
C13—C14	1.395 (4)		
			
C6—O1—C5	109.9 (2)	C15—C16—C17	117.0 (3)
C14—N4—C15	111.2 (2)	C15—C16—H16	121.5
C14—N4—C24	124.2 (2)	C17—C16—H16	121.5
C15—N4—C24	124.4 (2)	C16—C17—C18	121.2 (3)
N1—C1—C2	179.2 (4)	C16—C17—H17	119.4
C6—C2—C1	121.4 (3)	C18—C17—H17	119.4
C6—C2—C3	119.5 (3)	C17—C18—C19	120.7 (3)
C1—C2—C3	119.1 (3)	C17—C18—H18	119.7
N2—C3—C2	178.6 (4)	C19—C18—H18	119.7
C7—C4—C11	132.4 (3)	C20—C19—C18	118.3 (3)
C7—C4—C5	107.3 (2)	C20—C19—H19	120.9
C11—C4—C5	120.3 (3)	C18—C19—H19	120.9
O1—C5—C4	103.4 (2)	C15—C20—C19	120.3 (3)
O1—C5—C9	107.0 (2)	C15—C20—C21	109.1 (2)
C4—C5—C9	112.0 (3)	C19—C20—C21	130.6 (3)
O1—C5—C8	108.2 (2)	C20—C21—C14	101.6 (2)
C4—C5—C8	112.9 (2)	C20—C21—C22	109.6 (2)
C9—C5—C8	112.7 (3)	C14—C21—C22	112.6 (2)
O1—C6—C2	118.9 (3)	C20—C21—C23	110.4 (2)
O1—C6—C7	110.4 (2)	C14—C21—C23	111.2 (2)
C2—C6—C7	130.6 (3)	C22—C21—C23	111.1 (3)
C4—C7—C10	126.2 (3)	C21—C22—H22A	109.5
C4—C7—C6	108.8 (3)	C21—C22—H22B	109.5
C10—C7—C6	124.7 (3)	H22A—C22—H22B	109.5
C5—C8—H8A	109.5	C21—C22—H22C	109.5
C5—C8—H8B	109.5	H22A—C22—H22C	109.5
H8A—C8—H8B	109.5	H22B—C22—H22C	109.5
C5—C8—H8C	109.5	C21—C23—H23A	109.5
H8A—C8—H8C	109.5	C21—C23—H23B	109.5
H8B—C8—H8C	109.5	H23A—C23—H23B	109.5
C5—C9—H9A	109.5	C21—C23—H23C	109.5
C5—C9—H9B	109.5	H23A—C23—H23C	109.5
H9A—C9—H9B	109.5	H23B—C23—H23C	109.5
C5—C9—H9C	109.5	N4—C24—C25	113.6 (2)
H9A—C9—H9C	109.5	N4—C24—H24A	108.8
H9B—C9—H9C	109.5	C25—C24—H24A	108.8
N3—C10—C7	176.1 (3)	N4—C24—H24B	108.8
C12—C11—C4	125.4 (3)	C25—C24—H24B	108.8
C12—C11—H11	117.3	H24A—C24—H24B	107.7
C4—C11—H11	117.3	C26—C25—C24	115.3 (2)
C13—C12—C11	122.7 (3)	C26—C25—H25A	108.5
C13—C12—H12	118.7	C24—C25—H25A	108.5
C11—C12—H12	118.7	C26—C25—H25B	108.5
C12—C13—C14	125.9 (3)	C24—C25—H25B	108.5
C12—C13—H13	117.0	H25A—C25—H25B	107.5
C14—C13—H13	117.0	C25—C26—Br1	112.0 (2)
N4—C14—C13	122.2 (3)	C25—C26—H26A	109.2
N4—C14—C21	109.2 (2)	Br1—C26—H26A	109.2
C13—C14—C21	128.6 (3)	C25—C26—H26B	109.2
C20—C15—C16	122.5 (3)	Br1—C26—H26B	109.2
C20—C15—N4	108.9 (2)	H26A—C26—H26B	107.9
C16—C15—N4	128.5 (3)		
			
C6—O1—C5—C4	−3.6 (3)	C12—C13—C14—C21	2.0 (5)
C6—O1—C5—C9	114.8 (3)	C14—N4—C15—C20	1.6 (3)
C6—O1—C5—C8	−123.5 (3)	C24—N4—C15—C20	176.2 (2)
C7—C4—C5—O1	1.8 (3)	C14—N4—C15—C16	−177.6 (3)
C11—C4—C5—O1	−177.6 (3)	C24—N4—C15—C16	−3.0 (4)
C7—C4—C5—C9	−113.0 (3)	C20—C15—C16—C17	−0.1 (4)
C11—C4—C5—C9	67.6 (3)	N4—C15—C16—C17	179.0 (3)
C7—C4—C5—C8	118.5 (3)	C15—C16—C17—C18	0.2 (5)
C11—C4—C5—C8	−60.9 (4)	C16—C17—C18—C19	0.3 (5)
C5—O1—C6—C2	−176.3 (3)	C17—C18—C19—C20	−0.8 (5)
C5—O1—C6—C7	4.1 (3)	C16—C15—C20—C19	−0.5 (4)
C1—C2—C6—O1	175.3 (3)	N4—C15—C20—C19	−179.7 (3)
C3—C2—C6—O1	−6.1 (5)	C16—C15—C20—C21	179.2 (3)
C1—C2—C6—C7	−5.1 (5)	N4—C15—C20—C21	0.0 (3)
C3—C2—C6—C7	173.4 (3)	C18—C19—C20—C15	0.9 (5)
C11—C4—C7—C10	6.0 (5)	C18—C19—C20—C21	−178.7 (3)
C5—C4—C7—C10	−173.3 (3)	C15—C20—C21—C14	−1.4 (3)
C11—C4—C7—C6	179.7 (3)	C19—C20—C21—C14	178.3 (3)
C5—C4—C7—C6	0.5 (3)	C15—C20—C21—C22	−120.6 (3)
O1—C6—C7—C4	−2.9 (3)	C19—C20—C21—C22	59.1 (4)
C2—C6—C7—C4	177.6 (3)	C15—C20—C21—C23	116.7 (3)
O1—C6—C7—C10	171.0 (3)	C19—C20—C21—C23	−63.6 (4)
C2—C6—C7—C10	−8.6 (5)	N4—C14—C21—C20	2.3 (3)
C7—C4—C11—C12	3.6 (5)	C13—C14—C21—C20	−177.8 (3)
C5—C4—C11—C12	−177.2 (3)	N4—C14—C21—C22	119.4 (3)
C4—C11—C12—C13	−176.6 (3)	C13—C14—C21—C22	−60.7 (4)
C11—C12—C13—C14	178.5 (3)	N4—C14—C21—C23	−115.1 (3)
C15—N4—C14—C13	177.6 (3)	C13—C14—C21—C23	64.8 (4)
C24—N4—C14—C13	2.9 (4)	C14—N4—C24—C25	−76.2 (3)
C15—N4—C14—C21	−2.5 (3)	C15—N4—C24—C25	109.9 (3)
C24—N4—C14—C21	−177.1 (2)	N4—C24—C25—C26	−60.1 (4)
C12—C13—C14—N4	−178.1 (3)	C24—C25—C26—Br1	−63.5 (3)

### Hydrogen-bond geometry (Å, °)

**Table:**

*D*—H···*A*	*D*—H	H···*A*	*D*···*A*	*D*—H···*A*
C9—H9B···N1^i^	0.98	2.59	3.449 (5)	147
C23—H23B···Br1^ii^	0.98	2.99	3.962 (4)	171
C26—H26B···Br1^iii^	0.99	2.95	3.815 (4)	147

Symmetry codes: (i) *x*, *y*−1, *z*; (ii) *x*, *y*+1, *z*; (iii) −*x*+2, −*y*, −*z*+1.
